# The ectodomains of the lymphocyte scavenger receptors CD5 and CD6 interact with tegumental antigens from *Echinococcus granulosus sensu lato* and protect mice against secondary cystic echinococcosis

**DOI:** 10.1371/journal.pntd.0006891

**Published:** 2018-11-30

**Authors:** Gustavo Mourglia-Ettlin, Sebastián Miles, María Velasco-De-Andrés, Noelia Armiger-Borràs, Marcela Cucher, Sylvia Dematteis, Francisco Lozano

**Affiliations:** 1 Área Inmunología, Facultad de Química/Facultad de Ciencias, DEPBIO/IQB, Universidad de la República, Montevideo, Uruguay; 2 Immunoreceptors del Sistema Innat i Adaptatiu, Institut d'Investigacions Biomèdiques August Pi i Sunyer (IDIBAPS), Barcelona, Spain; 3 Instituto de Investigaciones en Microbiología y Parasitología Médica, Facultad de Medicina, Universidad de Buenos Aires, Buenos Aires, Argentina; 4 Servei d’Immunologia, Centre de Diagnòstic Biomèdic, Hospital Clínic de Barcelona, Barcelona, Spain; 5 Departament de Biomedicina, Universitat de Barcelona, Barcelona, Spain; IRNASA, CSIC, SPAIN

## Abstract

**Background:**

Scavenger Receptors (SRs) from the host’s innate immune system are known to bind multiple ligands to promote the removal of non-self or altered-self targets. CD5 and CD6 are two highly homologous class I SRs mainly expressed on all T cells and the B1a cell subset, and involved in the fine tuning of activation and differentiation signals delivered by the antigen-specific receptors (TCR and BCR, respectively), to which they physically associate. Additionally, CD5 and CD6 have been shown to interact with and sense the presence of conserved pathogen-associated structures from bacteria, fungi and/or viruses.

**Methodology/Principal findings:**

We report herein the interaction of CD5 and CD6 lymphocyte surface receptors with *Echinococcus granulosus sensu lato (s*.*l*.*)*. Binding studies show that both soluble and membrane-bound forms of CD5 and CD6 bind to intact viable protoscoleces from *E*. *granulosus s*.*l*. through recognition of metaperiodate-resistant tegumental components. Proteomic analyses allowed identification of thioredoxin peroxidase for CD5, and peptidyl-prolyl cis-trans isomerase (cyclophilin) and endophilin B1 (antigen P-29) for CD6, as their potential interactors. Further *in vitro* assays demonstrate that membrane-bound or soluble CD5 and CD6 forms differentially modulate the pro- and anti-inflammatory cytokine release induced following peritoneal cells exposure to *E*. *granulosus s*.*l*. tegumental components. Importantly, prophylactic infusion of soluble CD5 or CD6 significantly ameliorated the infection outcome in the mouse model of secondary cystic echinococcosis.

**Conclusions/Significance:**

Taken together, the results expand the pathogen binding properties of CD5 and CD6 and provide novel evidence for their therapeutic potential in human cystic echinococcosis.

## Author summary

Scavenger Receptors (SRs) are constituents of host’s innate immune system able to sense and remove altered-self and/or pathogen components. Data on their interaction with helminth parasites is scarce. In this work, we describe that CD5 and CD6 -two lymphoid SRs previously reported to interact with conserved structures from bacteria, fungi and viruses- recognize tegumental components in the cestode parasite *Echinococcus granulosus sensu lato (s*.*l*.*)*. Moreover, both receptors differentially modulate the cytokine release by host cells exposed to *E*. *granulosus s*.*l*. tegumental components. Importantly, the infusion of soluble forms of CD5 or CD6 improve infection outcomes in a murine model of secondary cystic echinococcosis. In summary, our results expand the pathogen binding properties of CD5 and CD6 and suggest their therapeutic potential against helminth infections.

## Introduction

The mammalian innate immune system relies on a limited number of germline-encoded and non-clonally distributed receptors for pathogen recognition, which have evolved to identify the so called pathogen associated molecular patterns (PAMPs): conserved microbial structures, essential for their survival and not shared by the host, such as lipopolysaccharide (LPS) from Gram-negative bacteria, lipotheichoic acid (LTA) from Gram-positive bacteria, lipoarabinomannan from mycobacteria, mannan from fungi, chitin from parasites, and viral RNA [1]. Such kind of receptors are collectively named pattern recognition receptors (PRRs), and can be grouped into structurally diverse classes according to the protein domains involved in pathogen recognition (e.g., C-type lectin domains or leucine-rich repeats) [1,2].

This is well exemplified by the Scavenger Receptors (SRs), a large group of cell surface and soluble protein receptors that are structurally diverse and participate in a wide range of biological functions (endocytosis, phagocytosis, adhesion, and signaling) following binding to multiple non-self or altered-self ligands [3,4]. Some SR (namely SR-A and SR-I) are characterized by the presence of one or multiple repeats of an ancient and highly conserved cysteine-rich protein domain named SRCR (for scavenger receptor cysteine-rich) and constitute a superfamily (SRCR-SF) comprising more than 30 different cell-surface and/or secreted proteins present from lower invertebrates to mammals, as well as in algae and plants [5,6]. Despite the high degree of structural conservation among SRCR-SF members, a common single unifying function has not been reported. However, a steadily growing bunch of SRCR-SF members is known to interact with diverse microbial (bacterial, fungal, parasitic and/or viral) structures [6,7]. This is the case of the functionally and structurally highly homologous lymphocyte SR-I receptors CD5 and CD6. These two receptors are encoded by contiguous genes thought to derive from duplication of a common ancestral gene and are mainly expressed on all T cells, and a minor subset of B cells (B1a cells) [6]. The extracellular regions of both receptors are exclusively composed of 3 consecutive SRCR domains showing extensive sequence identity [8]. Their diverging cytoplasmic tails are devoid of intrinsic catalytic activity but both display several structural motifs compatible with a signaling transduction function [6]. Importantly, CD5 and CD6 are physically associated with the clonotypic antigen-specific receptor complex present on T and B1a cells (TCR and BCR, respectively) [9,10] and are involved in the fine tuning of the activation and differentiation signals generated by such relevant receptors through still incompletely understood and complex signaling pathways [11]. In addition to their immunomodulatory properties, CD5 and CD6 also exhibit PRRs activities. Available data indicate that soluble and membrane-bound forms of CD6, but not of CD5, bind to Gram-negative and Gram-positive bacteria through recognition of LPS and LTA, respectively [12,13]. In contrast, soluble and membrane-bound forms of CD5, but not of CD6, recognize and bind to saprophytic and pathogenic fungal species through β-glucans [14]. More recently, CD5 has been reported as a key receptor for human hepatitis C virus (HCV) entry into T lymphocytes [15], and preliminary observations indicate that CD6 may interact with human immunodeficiency virus 1 (HIV-1) [16]. It remains to be explored, however, whether the broad PRR activity exhibited by CD5 and CD6 also includes other groups of pathogen besides bacteria, fungi and viruses.

Helminths -a diverse group of metazoan parasites able to produce long-lasting infections in immunocompetent hosts- currently affect one third of the world population [17]. Helminthiases are usually chronic infections due to the pathogens’ ability to adapt to the defense mechanisms triggered by infected hosts. Therefore, in most cases host immune responses are ineffective in parasite elimination, and are often associated with polarized and stereotyped Th2-type responses, with rare to no levels of Th1-type components [18]. In most helminthiases, such an early response bias does not associate with protective immunity [18–20], and therefore identification of innate receptors able to recognize and respond to parasite-derived components during early infection stages is highly relevant.

Among helminthiases, cystic echinococcosis (CE) -formerly known as hydatidosis- is a zoonotic disease caused by the larval stage of the cestode *Echinococcus granulosus sensu lato (s*.*l*.*)*, which shows a cosmopolitan distribution with high prevalence worldwide [21–23]. *E*. *granulosus s*.*l*. is composed of numerous variants initially called genotypes/strains (G1-G10), which nowadays are recognized as new species: *E*. *granulosus sensu stricto (s*.*s*.*)* (G1/G2/G3), *E*. *equinus* (G4), *E*. *ortleppi* (G5), *E*. *canadensis* (G6/G7/G8/G10) and *E*. *felidis* (‘lion strain’). Among them, the G1 genotype of *E*. *granulosus s*.*s*. is the most frequently found worldwide in livestock and humans [24]. Primary CE occurs in intermediate hosts (domestic and wild ungulates; accidentally humans) via ingestion of eggs containing oncospheres, which later develop into metacestodes -or hydatid cysts- mainly in the liver and lungs of the infected host. Secondary CE occurs after protoscolex (PSC) spillage from a fertile hydatid cyst within an infected intermediate host. This kind of CE derives from PSC developmental plasticity, which allows them to develop either into new cysts within intermediate hosts or into adult worms if ingested by a definitive host (usually dogs) [25]. Human secondary CE is an important medical problem associated with the surgical removal of primary cysts. In fact, although actual percentages of secondary CE cases post-surgery are debatable, recent studies have reported rates of 10–35% depending mainly on the type of surgery, the geographical location, and the follow-up time [26–28].

The murine model of secondary CE (inoculation of viable PSC into mice) has been widely used to study both the basic aspects of *E*. *granulosus s*.*l*. immunobiology [29–35], and the new chemotherapeutics or therapeutical protocols [36–38], novel vaccine candidates [39–41], and diagnostic or follow-up tools [42–44]. In this model, secondary CE can be divided into two stages: an early pre-encystment stage (until day 20–30 post-inoculation) with PSC developing into hydatid cysts [45], and a late or post-encystment stage in which differentiated cysts grow and eventually become fertile cysts [46]. Such a sequential developmental process is associated with a strong local control of inflammation during the initial phase of PSC differentiation into hydatid cysts [32,47].

The present report extends PRRs activities of both CD5 and CD6 receptors to helminth parasites, using *E*. *granulosus s*.*l*. as a case study. The data we provide indicate that ectodomains from both receptors recognize specific parasite components present in the tegument of PSC. Additionally, the prophylactic potential of CD5 and CD6 ectodomains infusion is shown using the murine model of secondary CE.

## Materials and methods

### Ethics statement

Experimental animal procedures were performed in compliance with the Spanish Animal Experimentation Ethics Committee of Universitat de Barcelona School of Medicine, and the Uruguayan Comisión Honoraria de Experimentación Animal (Universidad de la República) according to the Canadian Guidelines on Animals Care and the National Uruguayan Legislation No.18.611. Protocols were approved by Comité de Ética en el Uso de Animales (Facultad de Química—Universidad de la República) and were given the Protocol Approval Number 101900-000361-16 (www.expe.edu.uy).

### Mice

Wild-type Balb/c and C57BL/6N mice (8–12 weeks old female) were obtained from DILAVE-MGAP (Uruguay) or Charles River (France), and housed under specific pathogen-free (for *in vitro* studies) or conventional (for experimental infections) conditions at the animal facilities of Instituto de Higiene (Universidad de la República, Uruguay) and of Universitat de Barcelona School of Medicine (Spain). CD5-deficient (CD5^-/-^) (provided by C. Raman, University of Alabama, Birmingham, AL) [48] and CD6-deficient (CD6^-/-^) mice [49] on C57BL/6 background were maintained at the animal facilities of the Universitat de Barcelona School of Medicine (Spain) under specific pathogen-free conditions.

### Parasites and tegumental antigens isolation

For tegumental antigens extraction, *E*. *granulosus s*.*l*. PSC were obtained by aseptic puncture of fertile bovine hydatid cysts from Uruguayan abattoirs, washed several times with phosphate buffered saline (PBS) pH 7.2 containing gentamicin (40 μg/mL), and their viability assessed [29]. Tegumental proteins were extracted from PSC (viability ≥80%) using an extracting solution consisting of PBS plus 1% (w/v) MEGA-10, 5 mM EDTA, and 2 mM PMSF [39]. Briefly, 125,000 viable PSC/mL of extracting solution were incubated for 2 h at RT with gentle shaking. Then, PSC were allowed to settle down and the supernatant was removed and extensively dialyzed against PBS through a cellulose membrane (MW cut-off: 12,000 Da). Protein content of the obtained antigens (termed PSEx) was assessed using BCA Protein Assay Reagent (Pierce). PSEx were stored at -20°C until used. Treated PSC were washed thrice with PBS, and their physical integrity was confirmed by observation under a light microscope.

For experimental infections, *E*. *granulosus s*.*s*. PSC were obtained by aseptic puncture of fertile bovine or ovine hydatid cysts provided by Uruguayan abattoirs and Dr. Raúl Manzano-Román (IRNASA-CSIC, Salamanca, Spain), respectively. In both cases -Spanish as well as Uruguayan PSC- only parasite batches with ≥95% viability were used for experimental infections, and *E*. *granulosus s*.*s*. genotype was confirmed to belong to the G1 strain by sequencing a fragment of the gene coding for mitochondrial cytochrome c oxidase subunit 1 (CO1), as previously described [50].

### Expression, purification, and biotinylation of recombinant proteins

Production of purified recombinant soluble proteins encompassing the whole ectodomains of human CD5 (rshCD5; from R^25^ to D^345^) and CD6 (rshCD6; from D^25^ to R^397^) receptors (in PBS with 10% glycerol, pH 7.4) was performed based on previously reported methods [51] but using SURE CHO-M Cell line clones from the Selexis SURE-technology Platform (Geneva, Switzerland) and subjecting serum-free supernatants to size exclusion chromatography protocols developed at PX’Therapeutics (Grenoble, France). Bovine serum albumin (BSA) was from Sigma-Aldrich. Proteins were biotinylated with EZ-Link PEO-maleimide-activated biotin (Pierce) following the manufacturer’s instructions.

### PSC binding assays

Binding of biotin-labelled recombinant proteins to PSC was assessed according to [51] with slight modifications. Briefly, 5,000 PSC (viability ≥90%) were incubated in 600 μL of biotinylated rshCD5, rshCD6 or BSA protein solutions (20 μg/mL) in binding buffer (veronal buffer saline plus 5 mM CaCl_2_). After 1 h of incubation at RT with gentle orbital rotation, PSC were pelleted and washed thrice with binding buffer, and 125 μL of each solution was stored for further analyses. A new aliquot of 5,000 PSC was added to the remaining solutions and the same procedure was performed. Sequential incubations were performed with four PSC aliquots. Then, PSC pellets and 25 μL of stored supernatants (from the original solution and the last incubation), were mixed with SDS-PAGE reducing sample buffer and heat-denatured during 10 min at 100°C. Biotin-labelled proteins were developed by Western blotting (see below) following sample resolution in 12% SDS-PAGE and electro-transfer to PVDF membranes (Bio-Rad).

### ELISA binding assays

The binding ability of rshCD5 and rshCD6 proteins to PSC tegumental antigens was assessed by using 96-well microtiter plates (Nunc, Roskilde, Denmark) coated ON at 4°C with 100 μL/well of PSEx in PBS (10 μg/mL), and further blocked for 1 h at RT with 200 μL/well of PBS containing 1% (w/v) BSA. Increasing concentrations (0–40 μg/mL) of biotinylated rshCD5, rshCD6 or BSA (100 μL/well) were then added to the wells and incubated ON at 4°C. Bound protein was detected by the addition of 100 μL/well HRP-labelled streptavidin (1:5,000—Sigma) for 1 h at 37°C. Between every incubation step, unbound proteins were washed out thrice with PBS containing 0.05% (v/v) Tween-20. Enzymatic activity was developed at RT by adding 100 μL/well of 3,3’,5,5’-tetramethylbenzidine (TMB) substrate (Sigma). After stopping the reaction with H_2_SO_4_ 0.5 M (50μL/well), absorbance values were read at 450 nm.

To assess whether antigens recognized by rshCD5 and rshCD6 within PSEx were carbohydrates, a similar ELISA was performed including a step of PSEx oxidation with NaIO_4_ [33]. Briefly, PSEx-coated and BSA-blocked plates were incubated during 1 h with 20 mM NaIO_4_ in 50 mM acetate buffer pH 4.5 (200 μL/well) at RT. After three washings with acetate buffer, treated-wells were incubated for 30 min with 50 mM NaBH_4_ in PBS (250 μL/well), and 100 μL/well of biotin-labelled rshCD5 (20 μg/mL) or rshCD6 (10 μg/mL) was added to treated and untreated wells. The remaining ELISA protocol was performed as described above. Binding to NaIO_4_-resistant antigens was assessed as the percentage of absorbance values in treated wells respect to untreated wells.

ELISA competition was performed to explore potential overlapping between rshCD5 and rshCD6 for binding to PSEx. Briefly, PSEx-coated and BSA-blocked plates were incubated ON at 4°C with 100 μL/well of either a mixture made of a fixed amount of biotin-labelled rshCD5 (20 μg/mL) or rshCD6 (10 μg/mL) and increasing concentrations of unlabeled rshCD6 (0–40 μg/mL) or rshCD5 (0–20 μg/mL), respectively. After washing out unbound proteins, the remaining ELISA protocol was performed as described above. Ligand overlapping was assessed as the percentage of absorbance values in competed wells respect to non-competed wells (0 μg/mL of unlabeled protein).

### Two-dimension (2D) SDS-PAGE analyses

Analysis of PSEx by 2D SDS-PAGE was performed following standard protocols. Briefly, 300 μg of PSEx antigens were first precipitated by ON incubation at -20°C in 300 μL ice-cold acetone containing 20% of trichloracetic acid (TCA) and 0.07% dithiothreitol (DTT) to remove insoluble proteins and lipids. After centrifugation for 15 min at 10,000g and 4°C, the supernatant was discarded and 300 μL of ice-cold acetone containing 20% dimethyl sulfoxide (DMSO) and 0.07% DTT were added and incubated for 1 h at -20°C. Then, samples were centrifuged for 15 min at 10,000g and 4°C, the supernatants discarded, and 300 μL ice-cold acetone containing 0.07% DTT were added. This step was repeated twice. Finally, the pellet was lyophilized, rehydrated in immobilized pH gradient (IPG) buffer (GE Healthcare) and frozen at -80°C for 24 h to improve solubilization. For the first dimension, 7 cm linear pH gradient (pH 3–10) Immobilie DryStrips (GE Healthcare) were re-hydrated with the sample and run on an IPGphore isoelectric focusing system (9.5 h run and a total of 35.5 KV/h), and stored at -80°C until use. Strips were then soaked for 15 min in equilibration buffer (50 mM Tris-Cl pH 8.8, 6 M urea, 30% glycerol, 2% SDS, and traces of bromphenol blue) containing 10 mg/mL DTT, further soaked for 15 min in equilibration buffer containing 25 mg/mL iodoacetamide, and sealed to 10% acrylamide gels using 0.5% agarose in standard Tris-glycine electrophoresis buffer. Second dimension SDS-PAGE was run at 50 V for the first 15 min and then raised to 150 V until ending. Finally, replicates of 2D SDS-PAGE gels were subjected either to mass-spec compatible silver nitrate staining or to electro-transference to PVDF membranes (Bio-Rad) for Western blotting.

### Western blotting

Electro-transferred PVDF membranes either from PSC binding assays or from PSEx 2D SDS-PAGE were blocked with 1% (w/v, in PBS) BSA for 2 h at RT. Membranes from PSEx 2D SDS-PAGE were additionally incubated ON at 4°C with solutions of biotin-labeled rshCD5 or rshCD6 (15 μg/mL). All membranes were then incubated for 1 h at 37°C in a PBS solution of 0.1% (w/v) BSA and 0.05% (v/v) Tween-20 containing HRP-streptavidin (1:5,000—Sigma). Finally, membranes were extensively washed with PBS plus 0.05% (v/v) Tween-20, and blots were developed by chemo-luminescence (SuperSignal West Pico Substrate, ThermoScientific) in a G-Box equipment (Syngene).

### Proteomic analyses

Clean spots observed in PSEx 2D SDS-PAGE transferred PVDF membranes Western blotted with biotinylated rshCD5 and rshCD6 were manually back-mapped on gels for mass spectrometry identification at the Proteomic Facility of Pasteur Institut (Montevideo). Briefly, spots were excised, faded and tryptic digestions were performed using sequencing-grade modified trypsin (Promega). After gel extraction into 60% acetonitrile containing 0.1% TFA, the excess of acetonitrile was removed by speed vacuum. Peptide samples were then combined with an equal volume of matrix, spotted onto a MALDI sample plate, and allowed to dry at RT. Mass spectra were acquired on a 4800 MALDI TOF/TOF Mass Analyzer (Abi Sciex) operating in the positive ion reflector mode. Protein identifications were performed using an in-house Mascot v.2.3 search engine by searching a custom database that includes the full proteome of *E*. *granulosus s*.*l*. and *E*. *multilocularis*, composed of 20,787 sequences (10,310,548 residues) obtained from the Sanger Helminth Database. Additionally, every mass spectrum was also analyzed using NCBI database to discard possible host related proteins. The search criteria used were cystein carbamidomethylation and methionine oxidation as variable modification, and mass deviation <200 ppm with peptide fragment tolerance of 0.45 Da. Scores >56 were considered significant (*P*<0.05).

### Flow cytometry studies

Assessment of fluorescein isothiocyanate (FITC)-labelled PSEx binding to membrane-bound CD5 or CD6 was performed by flow cytometry analyses of parental 2G5 cells (a Jurkat cell derivative selected for deficient CD5 and CD6 expression [52]) and stable 2G5-transfectants expressing wild-type human CD5 (2G5-CD5.wt) or CD6 (2G5-CD6.wt) [10]. FITC labeling of PSEx was done as previously reported [53] using fluorescein isothyiocyanate (Sigma). Briefly, 1 mg of PSEx was dialyzed against 100 mM NaHCO_3_ buffer pH 9, and then 500 μg of FITC (Sigma) in DMSO were added. After 8 h of vigorous shaking in the dark, the mixture was extensively dialyzed against PBS and stored at 4°C until use. Binding assays were performed by incubating 2x10^5^ 2G5, 2G5-CD5.wt and 2G5-CD6.wt cells with increasing amounts of FITC-labelled PSEx in blocking buffer (PBS plus 10% human AB serum, 2% FCS and 0.02% NaN_3_) for 30 min at 4°C. Then, cells were washed thrice with washing buffer (PBS plus 2% FCS and 0.02% NaN_3_) and analyzed on a FACSCalibur flow cytometer using CellQuest software (Becton Dickinson). Additionally, competition assays were performed in a similar way, but incubating cells with a fixed amount (10 μg) of FITC-labelled PSEx in the presence of increasing amounts (5–20 μg) of unlabeled rshCD5, rshCD6 or BSA.

### Cell cultures

The influence of the interaction between PSC tegumental antigens and CD5/CD6 on the PSEx-induced cytokine profile was first assessed by cell cultures of spleen and peritoneal cells from naïve CD5^-/-^ [48], CD6^-/-^ [49] and their corresponding C57BL/6 wild-type littermates in the presence of increasing concentrations of PSEx (0–40 μg/mL). Secondly, peritoneal cells from naïve C57BL/6 wild-type mice were stimulated with a fixed concentration of PSEx (20 μg/mL) in the presence of increasing amounts of rshCD5, rshCD6 or BSA (0–40 μg/mL). Spleen cells were obtained by mechanically disrupting spleens with a syringe plunger through a cell strainer. Harvesting of peritoneal cells was done by repeated washings (4 times with 2 mL/washing) of peritoneal cavities with cold PBS plus 2% FCS. Both procedures were performed under sterile conditions. Cell pellets -either from spleen or peritoneal cells- were treated with red blood cell lysing buffer (Sigma) following manufacturer’s instructions, and then suspended in complete culture medium (RPMI 1640 plus 10% FCS, 50 μM 2-mercaptoethanol, 100 μg/mL streptomycin and 100 U/mL penicillin, all from Sigma) and counted. Cells were seeded in 96-wells U-bottom plates at 2x10^5^ cells/well in 200μL of complete culture medium and then incubated for 72 h at 37°C in a 5% CO_2_ atmosphere. Stimulation of cells with 10 μg/mL LPS (Sigma) was used as a positive control.

### Cytokines measurement by ELISA

Mouse cytokine levels in culture supernatants were determined by commercially available ELISA kits following the manufacturer’s instructions. The IL-17A ELISA kit was from R&D Systems. The IL-1β, IL-2, IL-4, IL-5, IL-6, IL-10, IL-12p40, TNF-α and IFN-γ BD OptEIA-Mouse ELISA Sets were from BD Biosciences Pharmigen.

### Murine model of secondary CE

To assess whether rshCD5 or rshCD6 could modulate CE outcome, secondary infections were performed in Balb/c mice [33,35]. Mice were administered with rshCD5, rshCD6 or BSA in 200 μL sterile PBS (25 μg, i.p.) one hour before (-1h) and after (+1h) i.p. inoculation of 2,000 PSC (viability ≥95%) in 200 μL of sterile PBS. Mice were euthanized 14 months post-challenge and peritoneal cysts were recovered. Groups were compared in terms of (*i*) frequency of infection (proportion of mice harboring at least one cyst), (*ii*) number of developed cyst within each mouse, and (*iii*) total mass of cyst within each mouse (cyst wet-weight).

### Statistical analyses

Depending on the characteristics of the values, statistical analyses were assessed by either Student’s t-test (parametric values), Mann-Whitney U-test (non-parametric values) or Fisher’s exact test (non-parametric contingencies). Differences were regarded as significant when *P* <0.05.

## Results

### CD5 and CD6 ectodomains bind to different PSC tegumental antigens

**Fig 1:**
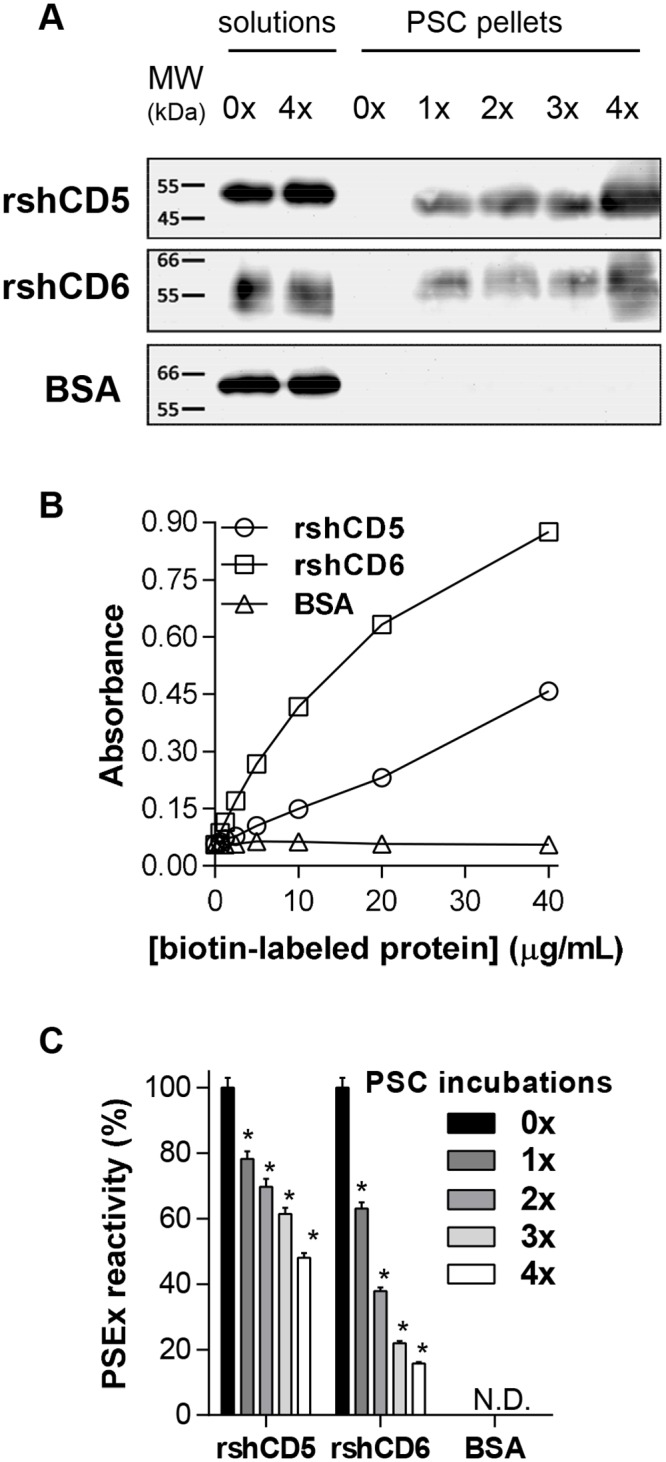
CD5 and CD6 ectodomains bind PSC tegumental antigens. (**A**) Biotin-labeled rshCD5, rshCD6 and BSA protein solutions (20 μg/mL) were sequentially incubated (4 times) with PSC suspensions (5,000 PSC; viability ≥90%), and pellets and solutions run on SDS-PAGE and further Western blotted with HRP-streptavidin. Lanes 1 and 2: protein solutions before (0x) and after 4 sequential incubations with PSC (4x), respectively. Lanes 3 to 7: PSC pellets after 0x to 4x sequential incubations. (**B**) ELISA assays showing the binding of increasing amounts of biotinylated rshCD5, rshCD6 and BSA proteins to PSEx-coated plates. (**C**) ELISA assays showing the binding of supernatants from sequential PSC incubations of biotin-labeled rshCD5, rshCD6 and BSA depicted in (**A**), to PSEx-coated plates. N.D. not detected. (*) Significant differences (Student’s t-test, *P* <0.05) respect to 0x results.

In order to determine whether the human CD5 and CD6 ectodomains are able to directly bind to the surface of viable *E*. *granulosus s*.*l*. PSC, pathogen-binding assays previously used for exploring their putative interaction with fungal and bacterial cell wall components, respectively, were performed [12,14]. Thus, biotinylated rshCD5 and rshCD6 proteins were sequentially incubated with viable PSC suspensions, and SDS-PAGE and Western blotting of pellets against streptavidin-HRP further assayed their adsorption to PSC. The results showed that both rshCD5 and rshCD6 (but not BSA, used as a negative control) bound to viable PSC (Fig 1A), indicating that they possess helminth-parasite binding activity.

Next, it was investigated whether the observed binding of rshCD5 and rshCD6 to viable PSC involved tegumental components. To that end, increasing concentrations of biotin-labeled proteins were assayed on ELISA plates coated with the antigenic fraction termed PSEx, composed of PSC tegumental antigens. Results depicted in Fig 1B show that both, biotinylated rshCD5 and rshCD6 (but not BSA), interact with structures present in the PSEx fraction in a dose-dependent manner. Additionally, when assayed the supernatants resulting from sequential incubations of PSC with biotin-labeled rshCD5 and rshCD6 depicted in Fig 1A, reactivity against PSEx decreased as the number of incubations increased, in accordance with a sequential co-precipitation phenomenon (Fig 1C).

**Fig 2:**
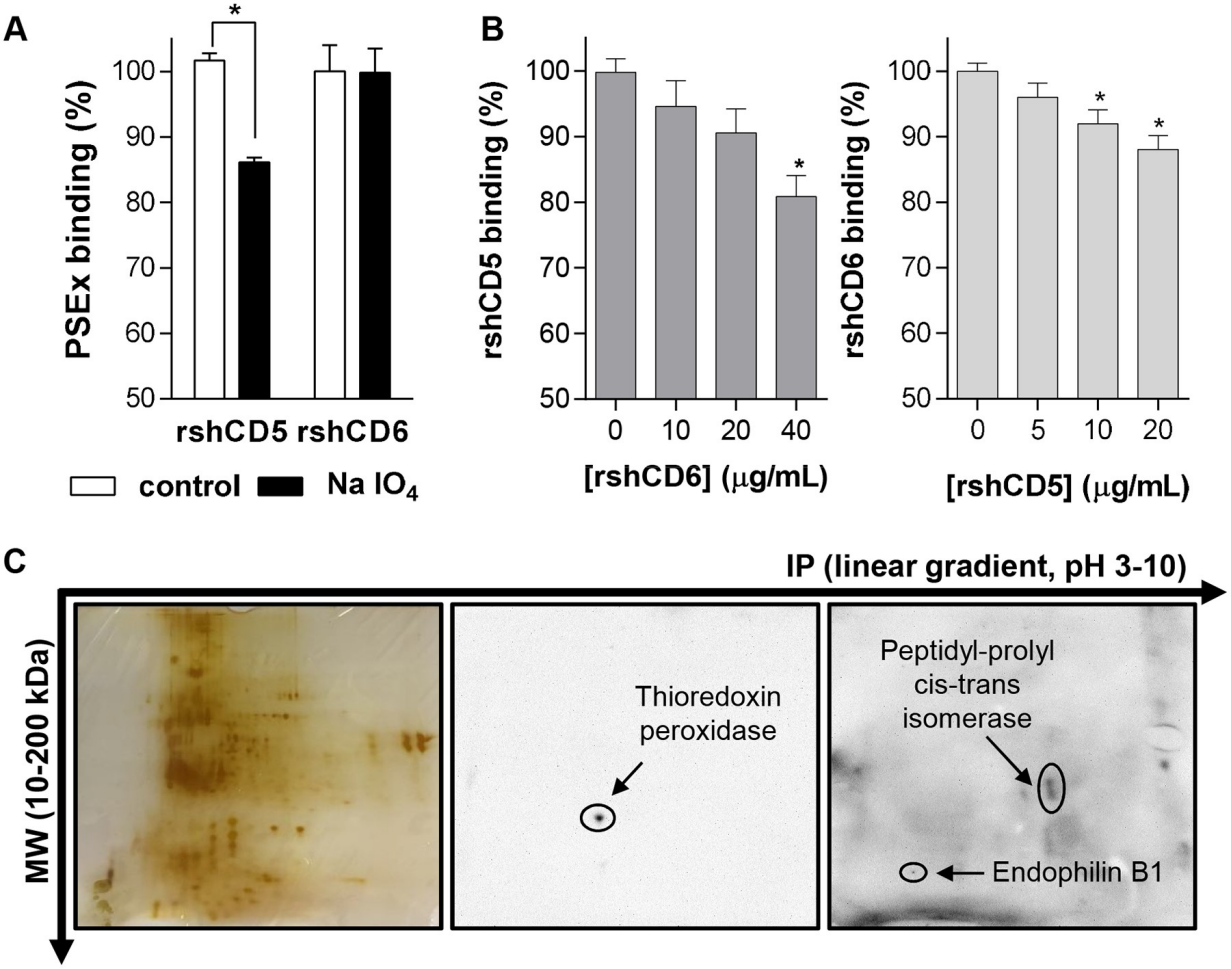
Tegumental antigens recognized by rshCD5 and rshCD6 are different. (**A**) ELISA assays showing binding of rshCD5 and rshCD6 to PSEx-coated plates undergoing metaperiodate treatment (NaIO_4_) or not (control). Binding to metaperiodate-resistant antigens was assessed as the percentage of absorbance values in treated wells respect to untreated wells (triplicates in each case). (**B**) Competition binding assays in which binding of a fixed amount of biotinylated rshCD5 (20 μg/mL; **left**) or rshCD6 (10 μg/mL; **right**) to PSEx-coated ELISA were competed with increasing amounts of unlabeled rshCD6 and rshCD5 proteins, respectively. Ligand overlapping was assessed (in triplicates) as the percentage of absorbance values in competed wells respect to non-competed wells (0 μg/mL of unlabeled protein). (**C**) 2D SDS-PAGE resolved PSEx fractions were either silver stained (**left**) or electro-transferred onto PVDF membranes for further Western blotting with biotin-labeled rshCD5 (**middle**) or rshCD6 (**right**). (*) Significant differences (Student’s t-test, *P* <0.05) respect to control (**A**) or uncompleted (**B**) wells.

Once evidenced the interaction of rshCD5 and rshCD6 with the PSEx fraction, the biochemical characterization of the PSEx components involved was addressed. To that end, an ELISA-based assay was first performed to determine whether rshCD5 and rshCD6 interactors were metaperiodate-sensitive (i.e. carbohydrates) or -resistant (i.e. proteins/lipids) compounds. Results depicted in Fig 2A indicate that all rshCD6- and most rshCD5-mediated interactions were metaperiodate-resistant, suggesting they are of protein and/or lipid nature. Then, in order to assess if the ligand patterns are similar or different for each molecule, we performed competition experiments in PSEx-coated ELISA plates with a fixed concentration of biotin-labeled rshCD5 incubated with increasing amounts of unlabeled rshCD6, and *vice versa* (i.e. fixed biotin-labeled rshCD6 and increasing amounts of unlabeled rshCD5). The results obtained indicate that rshCD5 and rshCD6 exhibit little overlapping regarding their PSEx interactions (Fig 2B). This was further supported by Western blotting the 2D SDS-PAGE resolved PSEx fraction with biotinylated rshCD5 or rshCD6 and HRP-labeled streptavidin. As illustrated by Fig 2C, rshCD5 and rshCD6 differed regarding their “spot” pattern reactive with the PSEx fraction. Accordingly, MALDI-TOF/TOF analyses identified parasite thioredoxin peroxidase as a potential interactor for rshCD5, and parasite peptidyl-prolyl cis-trans isomerase (cyclophilin) and endophilin B1 (antigen P-29) in the case of rshCD6 (Table 1). Summing up, these results indicate that both rshCD5 and rshCD6 molecules exhibit binding capacity to different structures -mainly proteins and/or lipids- present in the tegument of *E*. *granulosus s*.*l*. PSC, expanding their known spectrum of pathogen recognition.

**Table 1:** Potential parasite ligands for rshCD5 and rshCD6 identified by 2D SDS-PAGE and further MALDI-TOF/TOF mass spectrometry analysis.

Receptor	Potential ligand	UniProtKB identifier	MW (Da)	IP	Length (aa)	MASCOT score	Sequence coverage (%)	Previously identified
rshCD6	Endophilin B1	A0A068WMU2	28,782	5.83	252	245	67	74–76
	Peptidyl-prolyl cis-trans isomerase	P14088	17,343	6.41	162	232	35	73,75,78,79
rshCD5	Thioredoxin peroxidase	Q8T6C4	21,392	5.78	193	79	55	73–77

### Binding of membrane-bound CD5 and CD6 to PSC tegumental antigens

**Fig 3:**
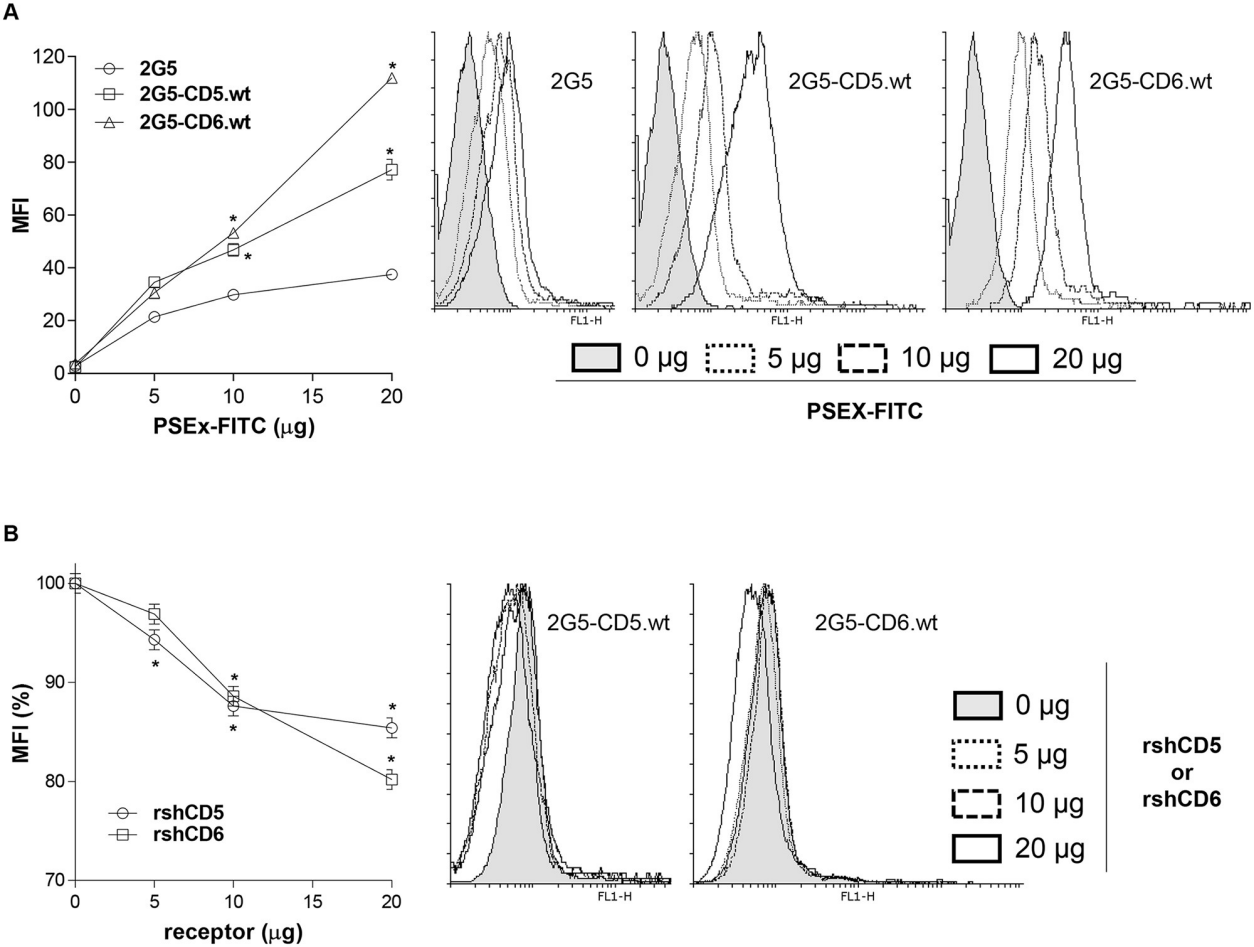
Native membrane-bound CD5 and CD6 receptors retain PSEx-binding capacity. (**A**) Flow cytometry analyses of 2G5, 2G5-CD5.wt or 2G5-CD6.wt cells stained with increasing amounts of FITC-labeled PSEx. Represented are the mean fluorescence intensity (MFI) values (**left**) and a representative flow cytometry histogram from each case (**right**). (**B**) Competition binding experiments in which 2G5-CD5.wt or 2G5-CD6.wt cells were stained with a fixed suboptimal amount of FITC-labeled PSEx in the presence or absence of different amounts of unlabeled rshCD5 or rshCD6 proteins. Both experiments were performed in quadruplicates and results are shown as mean +/- SD. (*) Significant differences (Student’s t-test, *P* <0.05) respect to 2G5 cells (**A**) or cells with 0 μg of competing proteins (**B**).

Next, it was questioned whether membrane-bound forms of human CD5 and/or CD6 lymphocyte receptors also retain their PSEx-binding activity. To that end, binding of FITC-labeled PSEx to parental 2G5 cells (a Jurkat T cell derivative selected for deficient CD5 and CD6 expression [52]) and to stable 2G5 transfectants expressing wild-type CD5 (2G5-CD5.wt) and CD6 (2G5-CD6.wt) surface receptors [10] was analyzed by flow cytometry. As shown in Fig 3A, mean fluorescence intensity (MFI) was significantly higher for 2G5-CD5.wt and 2G5-CD6.wt transfectants compared with parental untransfected 2G5 cells. The specificity of these interactions was confirmed by competition binding experiments, in which binding of a fixed amount of FITC-labeled PSEx to 2G5-CD5.wt and 2G5-CD6.wt cells was competed in a dose-dependent manner by unlabeled rshCD5 and rshCD6, respectively (Fig 3B). By contrast, unlabeled BSA (included as a negative control protein) did not inhibit binding of FITC-PSEx to 2G5-CD5.wt nor 2G5-CD6.wt (S1 Fig). Taken together, this evidence indicates that cell surface-expressed CD5 and CD6 retain PSEx-binding activity as well.

### Modulation of PSEx-induced cytokine production by membrane-bound and soluble CD5 and CD6 proteins

The influence of cell surface CD5 or CD6 expression on PSEx-induced cytokine production by spleen and peritoneal cells (PECs) from either naïve CD5^-/-^ or CD6^-/-^ mice, as well as their respective wild-type controls was first analyzed. To that end, cells were cultured for 72 h in the presence of increasing amounts of PSEx, and then cytokine production in supernatants was analyzed by capture ELISA. Spleen cells showed no significant PSEx-induced cytokine production over background (S2 Fig), in agreement to previous reports for other PSC-derived antigens [29,30,54]. By contrast, PSEx simulation of PECs resulted only in significant IL-10, TNF-α and IL-6 cytokine responses. Therefore, our further analyses focused on those cytokines within supernatants of cultured PECs. Since levels of spontaneous cytokine secretion usually differed between PECs from knockout and wild type mice (S3 Fig), results were further displayed in terms of fold changes for an easier interpretation.

**Fig 4:**
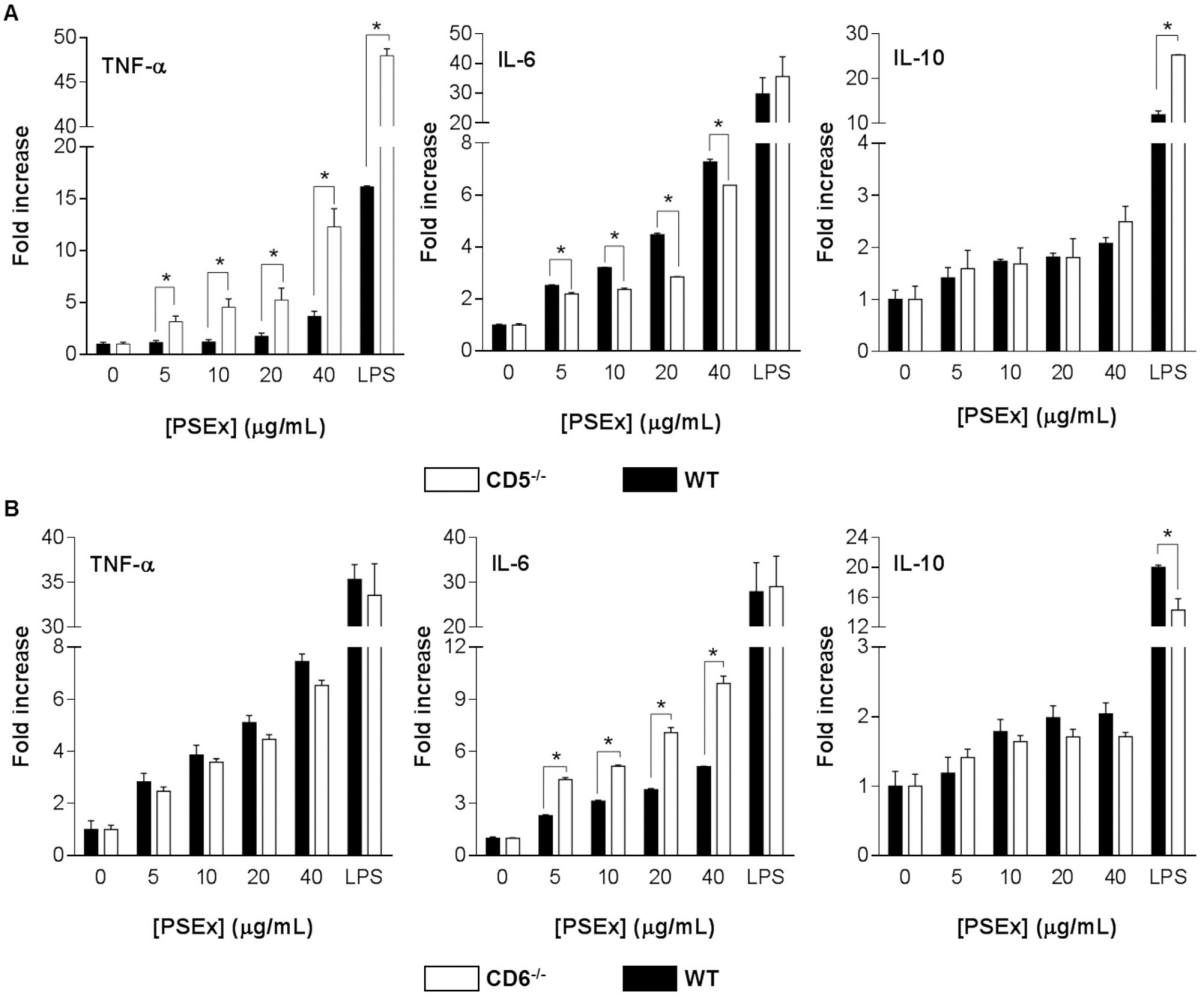
PSEx-induced cytokine production by naïve cells from CD5^-/-^ and CD6^-/-^ mice. (**A**) Peritoneal cells from naïve CD5^-/-^ (n = 3, pooled) and wild-type (n = 3, pooled) control mice, were cultured for 72 h in the presence of increasing amounts of PSEx, and then cytokine production in supernatants was analyzed by capture ELISA. Results are displayed as fold increases respect to unstimulated cells (mean +/- SEM). (**B**) Same as in (**A**) for CD6^-/-^ and wild-type control mice. Both experiments were performed in quadruplicates. (*) Significant differences (Student’s t-test, *P* <0.05) respect to unstimulated cells.

As illustrated by Fig 4A, while no differences were observed regarding IL-10 induction, PSEx-stimulated PECs from CD5^-/-^ mice underwent significant higher fold-increases for TNF-α and lower for IL-6 than their wild-type mice counterparts. Regarding CD6 surface expression, the results depicted in Fig 4B showed that PSEx-stimulation of PECs from CD6^-/-^ mice underwent significant higher fold-increases for IL-6 but not TNF-α or IL-10 than their wild-type mice counterparts. On the other hand, LPS-stimulated PECs (included as a positive stimulation control) from CD5^-/-^ and CD6^-/-^ mice exhibited higher fold-increases for IL-10 and TNF-α and lower fold-increases only for IL-10, respectively, than their wild-type counterparts (Fig 4A and 4B), suggesting an overall antigen-independent difference in stimulation threshold.

**Fig 5:**
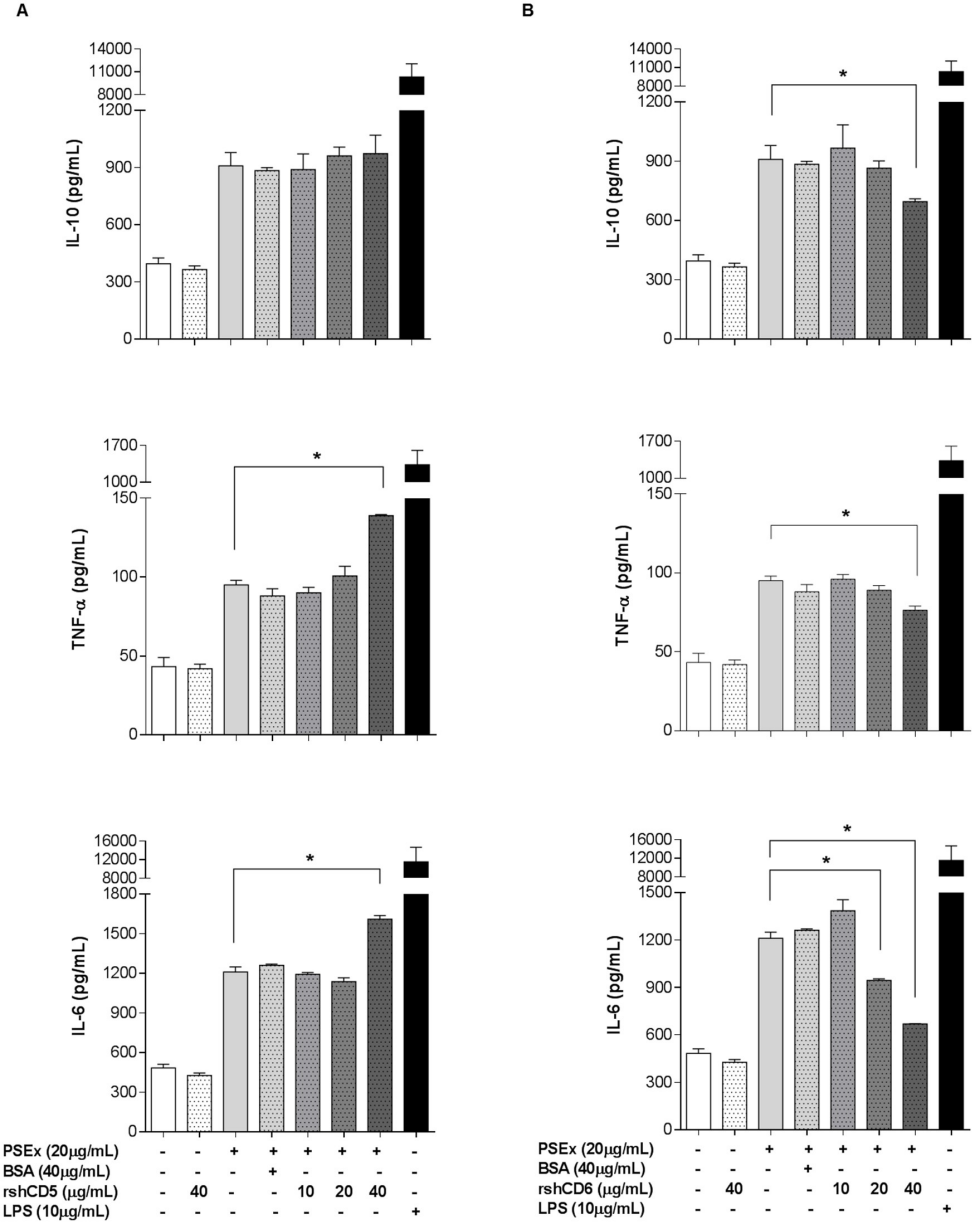
Modulation of PSEx-induced cytokine production by rshCD5 or rshCD6. (**A**) Peritoneal cells from naïve wild-type C57BL/6 mice (n = 3, pooled) were stimulated with a fixed concentration of PSEx (20 μg/mL) for 72 h in the presence of the indicated amounts of rshCD5, BSA or LPS. Results are displayed as cytokine concentration in supernatants (mean +/- SEM). (**B**) Same as in (**A**) using the indicated amounts of rshCD6 instead of rshCD5. Both experiments were performed in quadruplicates. (*) Significant differences (Student’s t-test, *P* <0.05) respect to cells stimulated only with PSEx.

To exclude possible CD5/CD6-independent alterations in knockout mice, TNF-α, IL-6 and IL-10 cytokine levels in supernatants of PSEx-stimulated PECs from wild-type C57BL/6 mice in the presence of increasing amounts of rshCD5 or rshCD6 were also assessed. This strategy might reduce the interaction of PSEx with membrane-bound CD5 and CD6 through direct competition with the soluble forms of the receptor recombinant ectodomains. Regarding PSEx-induced IL-10 secretion, no variations due to rshCD5 addition was observed, while rshCD6 significantly reduced IL-10 levels (Fig 5A and 5B, top bar charts). On the other hand, rshCD5 as well as rshCD6 both modified TNF-α and IL-6 (Fig 5A and 5B, middle and bottom bar charts) production in response to PSEx, but in opposite ways. Thus, while rshCD5 addition increased PSEx-induced TNF-α and IL-6 production by wild-type PECs, rshCD6 decreased the secretion levels of both cytokines. BSA addition (included as a negative control) did not affect PSEx-stimulated cytokine responses (Fig 5A and 5B).

Taken together this set of results indicates that either the absence of cell surface CD5 and CD6 receptors or the presence of both receptors in soluble form modulate the cytokine responses induced by tegumental antigens from *E*. *granulosus s*.*l*. PSC in different ways. Interestingly, the blockade of PSEx components by rshCD5 seems to up-regulate pro-inflammatory cytokine responses (i.e. increasing TNF-α and IL-6 secretion, without affecting IL-10 production levels), while blockade through rshCD6 seems to overall down-regulate the PSEx-induced cytokine response (i.e. decreasing the production of the three induced cytokines).

### Infusion of rshCD5 or rshCD6 protects mice from secondary CE

**Fig 6:**
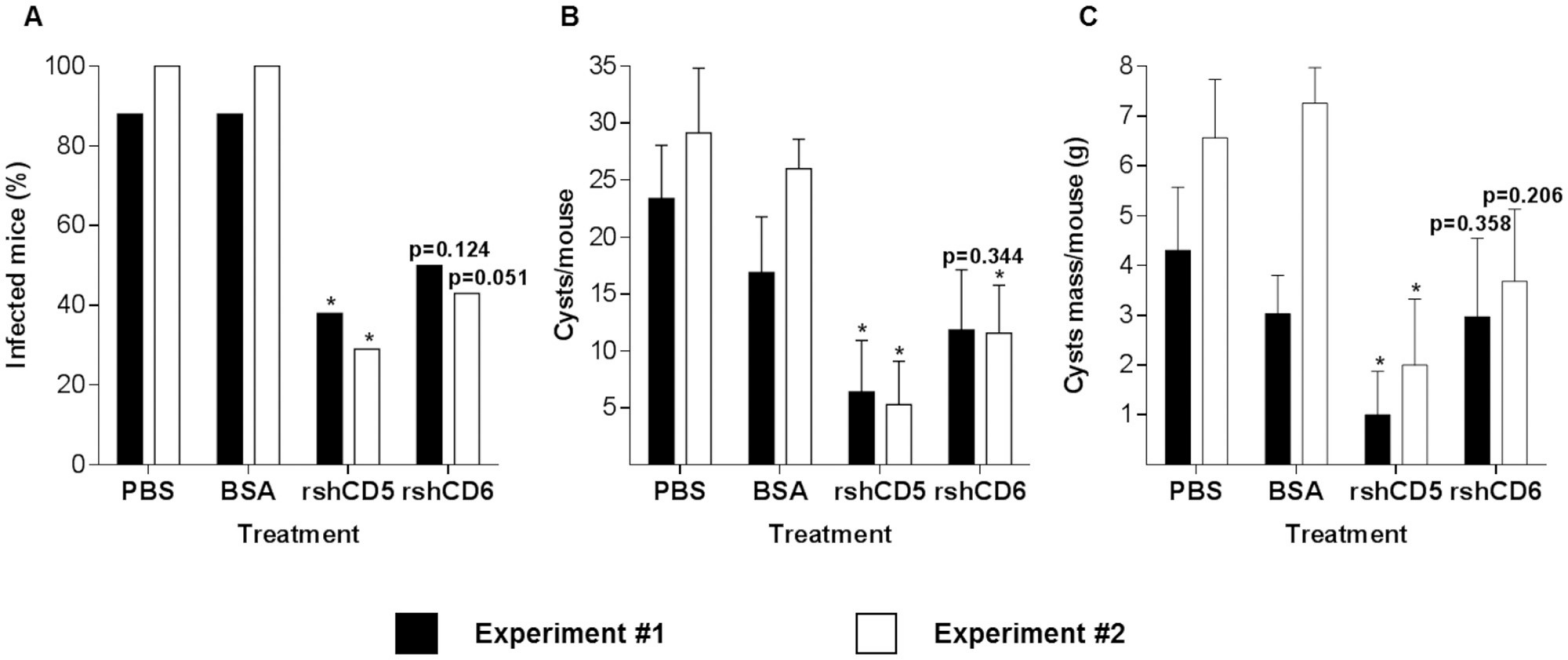
Infusion rshCD5 or rshCD6 protects mice from secondary CE. The anti-parasite prophylactic potential of rshCD5 or rshCD6 was assessed in the murine model of secondary CE by infusing PBS, rshCD5, rshCD6 or BSA (25μg/dose/mouse, i.p.) 1 h before and 1 h after i.p. inoculation of 2,000 highly viable PSC/mouse into Balb/c mice (n = 6–8 mice per experimental group). At 14 months post infection, mice were euthanized, and the peritoneal hydatid cysts within each mouse were counted and weighted. Results are displayed as percentage of infected mice (**A**), number (mean +/- SEM) of hydatid cysts per mouse (**B**), and total wet weight (mean +/- SEM) of hydatid cysts per mouse (**C**). The experiment was performed twice. (*) Significant differences (*P* <0.05) respect to the PBS group were assessed by either Fisher’s exact test (**A**) or Mann-Whitney U-test (**B** and **C**).

In light of the observed modulation by rshCD5 and rshCD6 of PSEx-induced cytokine responses in PECs from wild-type mice, it was further assessed whether rshCD5 or rshCD6 administration would modify the infection outcome in a mouse model of secondary CE. To that end, rshCD5 and rshCD6 (25 μg/mouse) were i.p. infused 1 h before and after i.p. inoculation of viable PSC (2,000/mouse) into Balb/c mice, a highly susceptible mouse strain to secondary CE [33]. The i.p. route for rshCD5/rshCD6 administration was chosen because the peritoneal cavity is the natural anatomical site for infection establishment and hydatid cyst development in this infection model. At 14 months post-infection, mice were euthanized for infection inspection, and the peritoneal hydatid cysts within each mouse were counted and weighted. As illustrated by Fig 6A, rshCD5 infusion exhibited a remarkable prophylactic potential against secondary CE, since it significantly reduced the proportion of infected individuals, as well as the number of hydatid cysts per mouse (Fig 6B), and the total wet weight of hydatid cysts per mouse (Fig 6C). On the other hand, rshCD6 infusion also exhibited some degree of prophylactic potential in secondary CE, since a trend towards reduction in the proportion of infected mice (Fig 6A) and the number of hydatid cysts per mouse (Fig 6B) was observed. Infusion of equivalent doses of BSA did not affect any parasitological parameter of infection outcome (Fig 6A–6C).

Finally, our results showed that rshCD5 -and to a lesser extent rshCD6 as well- exhibit prophylactic potential in the murine model of secondary CE.

## Discussion

Effective mammalian immune responses rely on the early recognition of pathogen-derived components by innate immunity related receptors, otherwise named PRRs. Data on key early steps of helminth parasitic infections is scarce. Functional approaches suggest the involvement of different TLRs (namely TLR4, TLR3, and TLR2) [55–59], and SRs in the recognition of helminth components. Current mammalian SRs include 10 different classes (SR-A to SR-L; excluding SR-C only present in *Drosophila melanogaster*), being class E (SR-E) the most important group in helminth-derived antigens recognition, including Dectin-2 [60], Mannose Receptor/CD206 [61–63], CLEC4F/CLECSF13 [64], and DC-SIGN/CD209a [65]. Our work expands the group of SRs interacting with helminth pathogens to CD5 and CD6, two lymphoid members of the class I (SR-I).

SR-I ectodomains harbor several tandem repeats of the SRCR protein module. In addition to CD5 and CD6, SR-I members include CD163A/M130, CD163B/M160, SCART1, SCART2 and WC1 [3,4]. Macrophages and lymphocyte subsets expressing CD163 or WC1, respectively, play a role against certain parasite infections (e.g. *Theileria parva* [66], *Leishmania braziliensis* [67], *Trypanosoma vivax* [68], or *Neospora caninum* [69]), but no direct interaction with parasite helminth structures has been previously reported.

A single C-terminal SRCR domain characterizes class A SRs (SR-A), including the SR-AI, MARCO, and SCARA5 receptors [3]. While some evidence supports the involvement of SR-AI and MARCO in parasite infection (*Schistosoma japonicum* and *Leishmania major*, respectively) [70,71], the ability of SR-AI to directly recognize helminth components has only been shown for *Heligmosomoides polygyrus* calreticulin [72]. The present study shows that CD5 and CD6 should be added to the list of SRs able to sense helminth components. Soluble CD5 and CD6 physically bind to different components within the tegument of *E*. *granulosus s*.*l*. PSC, modulate their induced cytokine profiles in naïve peritoneal cells, and protect mice from secondary CE.

Tegumental components are crucial for helminth physiology (i.e. nutrients up-take and waste disposal) and for their ability to modulate immune responses leading to chronic parasite establishments. They are the first parasite structures recognized by soluble and/or membrane-bound host receptors. Therefore, it is highly relevant to identify which receptors hold the ability to recognize them and, if possible, the involved parasite structures. We determine that human CD5 and CD6 ectodomains bind to the surface of viable PSC (Fig 1A). More specifically, CD5 and CD6 interact with components of an antigenic fraction termed PSEx, which is mainly composed of tegumental antigens from PSC (Fig 1B). Such PSEx components are metaperiodate-resistant compounds, indicating their protein and/or lipid nature (Fig 2A). Additionally, bound components differed depending on the receptor analyzed (Fig 2B). 2D SDS-PAGE and MALDI-TOF/TOF analyses of the PSEx fraction provides a differentiated spot pattern (Fig 2B and 2C), and a distinctive set of potential parasite ligands for each receptor: thioredoxin peroxidase for CD5, and peptidyl-prolyl cis-trans isomerase (cyclophilin) as well as endophilin B1 (P-29 antigen) for CD6 (Table 1). These results show that CD5 and CD6 ligands within PSEx do not fully overlap.

The CD5 and CD6 ligands besides being present in the PSEx fraction, have been found in other sources of *E*. *granulosus s*.*l*. antigens. Thioredoxin peroxidase has been detected in different PSC antigen sources [73–75], in hydatid fluid [76], in adult worms [74], and in extracellular vesicles obtained from fertile cysts [77]. Endophilin B1 (P-29 antigen) has been detected in somatic antigens of PSC and adult worms [74], in nuclear and cytosolic extracts of PSC [75], and in hydatid fluid [76]. Finally, peptidyl-prolyl cis-trans isomerase (cyclophilin), has been detected in PSC excretion/secretion products [73], in nuclear and cytosolic extracts of PSC [75], and in hydatid fluid [78,79]. Implying that CD5/CD6 parasite sensing would span the different stages of *E*. *granulosus s*.*l*. life cycle and not be limited to tegumental structures from PSC.

PSEx interaction with CD5 and CD6 is also extendable to their membrane-bound forms, as demonstrated by binding (and competition binding) experiments of FITC-labelled PSEx to parental and stably CD5- and CD6-transfected 2G5 cells -a Jurkat cell derivative deficient for surface CD5 and CD6 expression (Fig 3A and 3B). The basal interaction observed between PSEx and parental 2G5 cells suggests other cell surface receptors in parasite interaction (Fig 3A). Jurkat cells (a human CD4^+^ T cell lymphoma line) have been shown to express most TLRs [80], and 2G5 cell line in particular has been previously checked for surface expression of TLR2 and TLR4 [12,14]. Therefore, membrane-expressed CD5 and CD6 receptors specifically interact with PSEx components, even if other surface molecules may concomitantly act as PSEx-binding receptors.

Specific recognition of parasite structures by membrane-bound CD5 and CD6 may have relevant functional consequences, since both receptors induce intracellular signalling (namely MAPK cascade activation) when ligated by PAMPs [12,14]. PECs harbor CD5 and/or CD6 expressing immune cells involved in helminth infection protection. They include all T cells (comprising Tγδ and iNKT) and B1a cells as well as a macrophage subset [81]. Interestingly, CD5^+^ B1a cells are an important source of polyreactive natural IgM antibodies [82] and of IL-10 [83]. Thus, CD5- and CD6-mediated signalling by immune PECs may contribute to relevant biological effects, like modulation of cytokine production and release. Accordingly, PSEx-induced stimulation of PECs from CD5- and CD6-deficient mice resulted in different cytokine responses compared with their wild type controls (Fig 4). Interestingly, such differences were also observed following LPS-stimulation, suggesting an overall difference in stimulation thresholds (Fig 4). Indeed, data from knockout mice have shown the involvement of membrane-bound CD5 and CD6 in the fine-tuning of T (and likely B1a) cell subset responses [11,84]. Thus, the possibility that differences observed in PSEx-induced cytokine production by CD5- and CD6-deficient mice could be due to effects beyond direct recognition of parasite ligands by membrane-bound CD5 or CD6, was further excluded by similar PSEx stimulation studies in wild type mice in the presence of increasing amounts of soluble CD5 or CD6. The results showed that rshCD5 up-regulated PSEx-induced pro-inflammatory cytokine responses (i.e. increased TNF-α and IL-6 secretion, without altering IL-10 production rates) (Fig 5A), while rshCD6 down regulated the overall (TNF-α, IL-6 and IL-10) cytokine response (Fig 5B). Such findings are highly relevant either in a basic immunological sense, as well as from a potential prophylactic point of view.

Cytokine profiles are relevant for host susceptibility/resistance to *E*. *granulosus s*.*l*. infection. Thus, pro-inflammatory responses have been associated with host protection either in experimental infection models [30,34,85,86] as well as in human patients [87–91], being nitric oxide-mediated mechanisms involved in such protection [89–92]. Our results indicate that CD5 or CD6 binding to tegumental antigens from PSC contribute to cytokine induction associated with parasite establishments, and is not optimal for parasite killing and clearance. From a prophylactic point of view, *in vivo* infusion of rshCD5 or rshCD6 during the early stages of parasite establishment may be a useful strategy for immunomodulating the host into an anti-parasite state. Accordingly, the assessment of *in vivo* administration of rshCD5 or rshCD6 in a mouse model of secondary CE resulted in a remarkable prophylactic potential of the former, since it reduced not only the proportion of infected mice, but also the number of developed hydatid cysts per mouse and their parasite loads (Fig 6). A trend towards reduction in the proportion of infected individuals and the number of developed hydatid cysts was observed in rshCD6 infusion (Fig 6). The prophylactic potential of rshCD5 may correspond to pro-inflammatory cytokine (TNF-α and IL-6) upregulation (Fig 5A), while CD6 lesser efficiency parallels down-modulating cytokine production, especially IL-10 (Fig 5B), a cytokine usually associated with increased susceptibility to *E*. *granulosus s*.*l*. infection [31,34,90]. In this sense, our preclinical results might be useful for designing/proposing a novel strategy to reduce secondary infection rates in CE patients. For example, once a hydatid cyst is surgically removed, a concomitant intraperitoneal infusion of rshCD5 -or rshCD6- would help in preventing remaining PSC to develop into new hydatid cysts.

Interestingly, besides modulating cytokine production, the potential ligands identified for both CD5 and CD6 receptors are highly relevant for *E*. *granulosus s*.*l*. physiology. Thioredoxin peroxidase is a key enzyme for reactive oxygen species detoxification in *E*. *granulosus s*.*l*. [93,94]; while peptidyl-prolyl cis-trans isomerase (cyclophilin) has been associated with parasite sensitivity to lethal effects of cyclosporine A [95], and endophilin B1 (P-29 antigen) revealed significant protective activity against secondary CE in mice [96], as well as against primary infection in sheep [97], when used as a vaccine antigen. Therefore, the binding of such parasite components by rshCD5 or rshCD6 may also contribute to a better host parasite control.

In conclusion, we provide the first evidence for direct recognition of tegumental PSC structures from *E*. *granulosus s*.*l*. by SR-I CD5 and CD6, in addition to bacterial, fungal, and/or viral components binding. Moreover, we prove the prophylactic potential of soluble CD5 (and CD6) infusion in the mouse model of secondary CE. In this sense, such a prophylactic potential could be ascribed either to: *(i)* a cytokine-modulating activity through the competition with the interaction between tegumental antigens and host membrane-bound forms of CD5/CD6; *(ii)* the blockade of key tegumental components highly relevant for PSC physiology; or *(iii)* a mixture of both situations finally contributing to a better parasite control. Additionally, although CE has a cosmopolitan distribution and represents a major public health problem in regions of South America, Mediterranean, Central and Western Asia, and East Africa [98], our PSC experimental infections were performed with *E*. *granulosus s*.*s*. G1 genotype, which shows the highest cosmopolitan distribution and is responsible for most human CE cases worldwide [24,99]. Further work is required to ascertain CD5 and CD6 prophylactic potential in other helminth-driven pathologies and to explore if additional SRCR-SF members share such interactions.

## Supporting information

S1 Fig Native membrane-bound CD5 and CD6 receptors retain PSEx-binding capacity. Competition binding experiments in which 2G5-CD5.wt or 2G5-CD6.wt cells were stained with a fixed suboptimal amount of FITC-labeled PSEx in the presence or absence of different amounts of unlabeled BSA. Both experiments were performed in quadruplicates and results are shown as mean +/- SD. (*) Significant differences (Student’s t-test, *P* <0.05) respect to cells with 0 μg of competing BSA.
(TIF)

S2 Fig PSEx-induced cytokine production by naïve spleen cells from CD5^-/-^, CD6^-/-^ and their WT littermates. Spleen cells from naïve CD5^-/-^ or CD6^-/-^ (n = 3, pooled) and their respective wild-type (n = 3, pooled) control mice, were cultured for 72 h in the presence of increasing amounts of PSEx or LPS (positive control), and then cytokine production in supernatants was analyzed by capture ELISA. Cytokine concentrations are displayed as mean +/- SD of quadruplicates. ND (Not Detected).
(TIF)

S3 Fig Basal cytokine production by peritoneal cells from CD5^-/-^, CD6^-/-^ and their respective WT mice. Peritoneal cells from naïve CD5^-/-^, CD6^-/-^ and their respective wild-type control mice (in all cases: n = 3, pooled), were cultured for 72 h in complete culture medium alone, and then IL-6, IL-10 and TNF-α production in supernatants was analyzed by capture ELISA. Cytokine concentrations are displayed as mean +/- SD of quadruplicates. (*) Significant differences (Student’s t-test, *P* <0.05) respect to the corresponding WT cells.
(TIF)
