# Supplementary figures and images for: The ectodomains of the lymphocyte scavenger receptors CD5 and CD6 interact with tegumental antigens from Echinococcus granulosus sensu lato and protect mice against secondary cystic echinococcosis
Source: PLoS Negl Trop Dis. 2018 Nov 30;12(11):e0006891. doi: 10.1371/journal.pntd.0006891 (PMC6267981; doi:10.1371/journal.pntd.0006891)

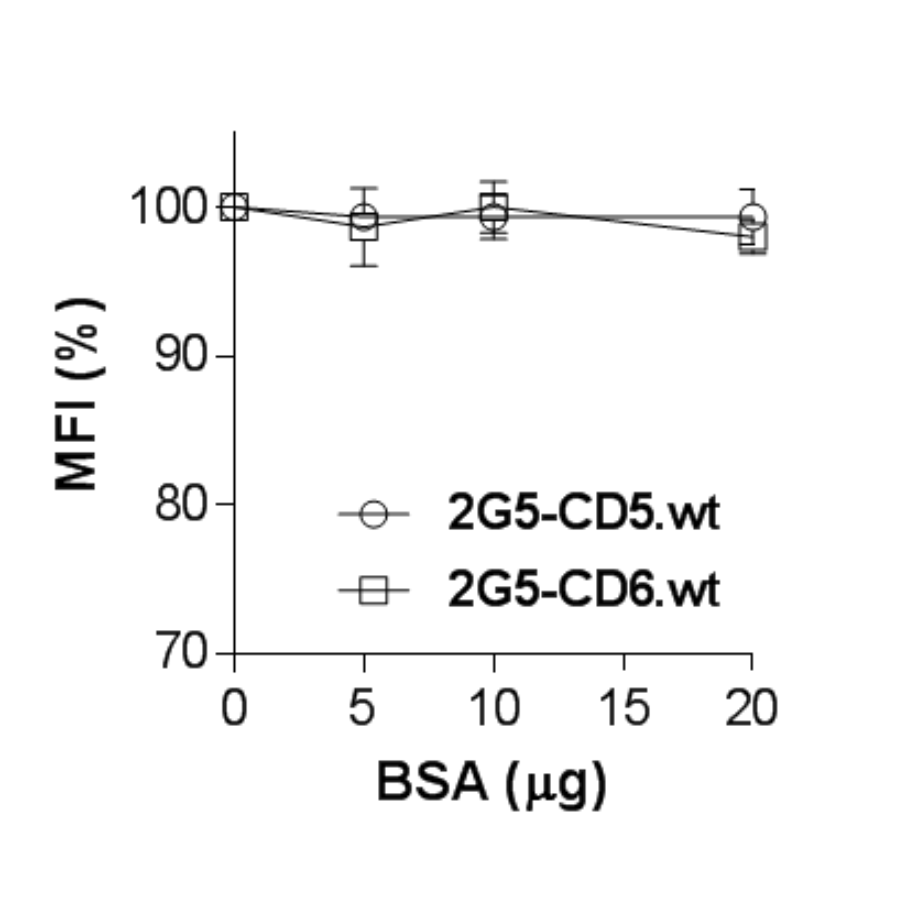

Supplement: S1 Fig — Competition binding experiments in which 2G5-CD5.wt or 2G5-CD6.wt cells were stained with a fixed suboptimal amount of FITC-labeled PSEx in the presence or absence of different amounts of unlabeled BSA. Both experiments were performed in quadruplicates and results are shown as mean +/- SD. (*) Significant differences (Student’s t-test, P <0.05) respect to cells with 0 μg of competing BSA. (TIF) [file pntd.0006891.s001.tif]

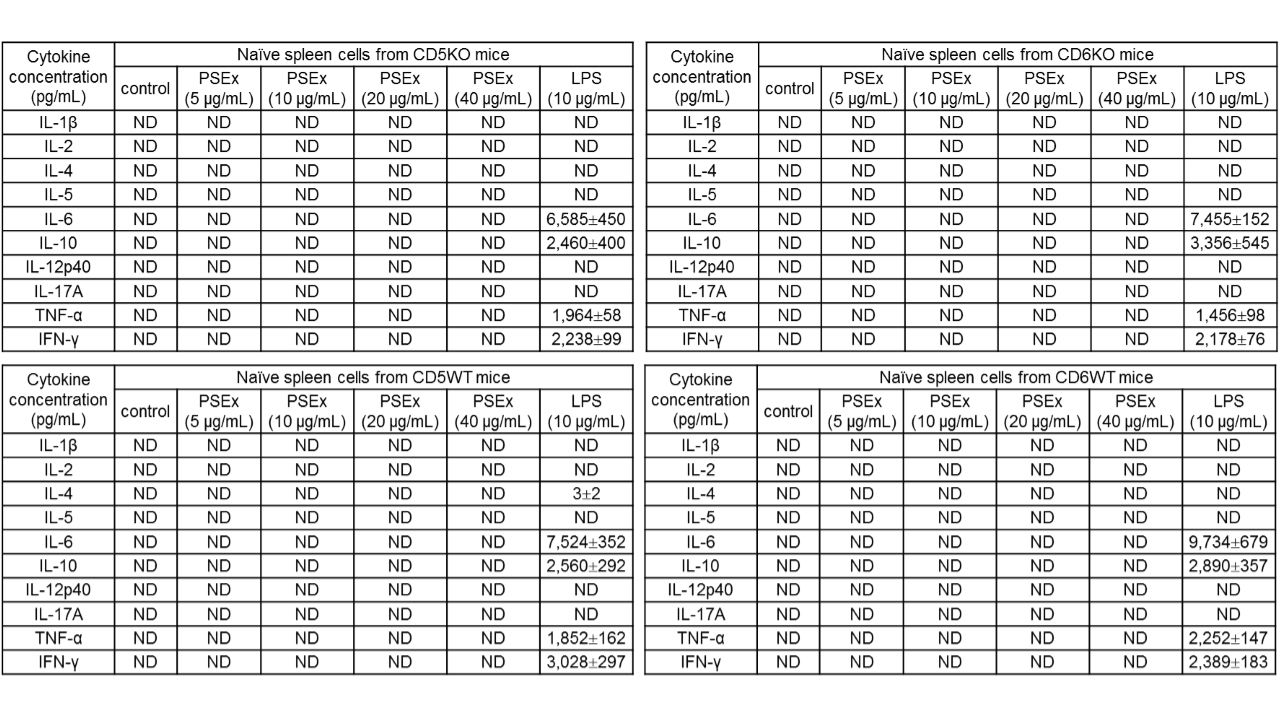

Supplement: S2 Fig — Spleen cells from naïve CD5-/- or CD6-/- (n = 3, pooled) and their respective wild-type (n = 3, pooled) control mice, were cultured for 72 h in the presence of increasing amounts of PSEx or LPS (positive control), and then cytokine production in supernatants was analyzed by capture ELISA. Cytokine concentrations are displayed as mean +/- SD of quadruplicates. ND (Not Detected). (TIF) [file pntd.0006891.s002.tif]

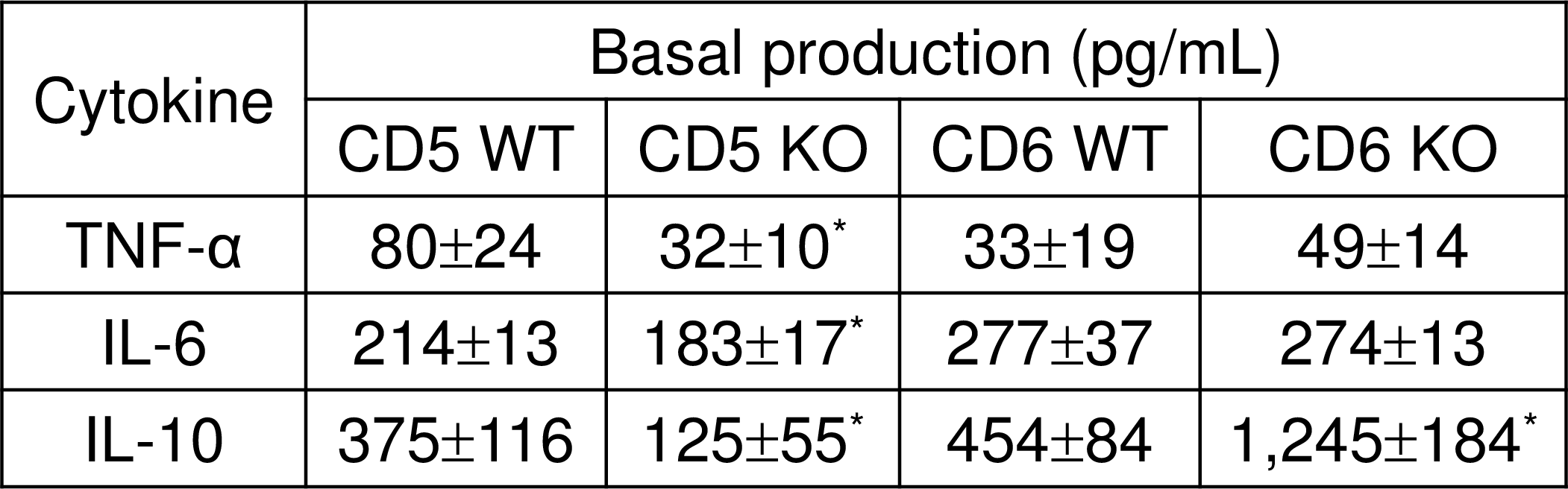

Supplement: S3 Fig — Peritoneal cells from naïve CD5-/-, CD6-/- and their respective wild-type control mice (in all cases: n = 3, pooled), were cultured for 72 h in complete culture medium alone, and then IL-6, IL-10 and TNF-α production in supernatants was analyzed by capture ELISA. Cytokine concentrations are displayed as mean +/- SD of quadruplicates. (*) Significant differences (Student’s t-test, P <0.05) respect to the corresponding WT cells. (TIF) [file pntd.0006891.s003.tif]
